# Accelerating a cross-correlation score function to search modifications using a single GPU

**DOI:** 10.1186/s12859-018-2559-6

**Published:** 2018-12-12

**Authors:** Hyunwoo Kim, Sunggeun Han, Jung-Ho Um, Kyongseok Park

**Affiliations:** 1 Research Data Hub Center, Korea Institute of Science and Technology Information, Daejeon, 34141 Republic of Korea; 2 KISTI Scientific Data School, Korea Institute of Science and Technology Information, Daejeon, 34141 Republic of Korea; 3 Super Computing Cloud Center, Korea Institute of Science and Technology Information, Daejeon, 34141 Republic of Korea

**Keywords:** Peptide identification, Tide, Cross-correlation score function, High performance computing, PTM search

## Abstract

**Background:**

A cross-correlation (XCorr) score function is one of the most popular score functions utilized to search peptide identifications in databases, and many computer programs, such as SEQUEST, Comet, and Tide, currently use this score function. Recently, the HiXCorr algorithm was developed to speed up this score function for high-resolution spectra by improving the preprocessing step of the tandem mass spectra. However, despite the development of the HiXCorr algorithm, the score function is still slow because candidate peptides increase when post-translational modifications (PTMs) are considered in the search.

**Results:**

We used a graphics processing unit (GPU) to develop the accelerating score function derived by combining Tide’s XCorr score function and the HiXCorr algorithm. Our method is 2.7 and 5.8 times faster than the original Tide and Tide-Hi, respectively, for 50 Da precursor tolerance. Our GPU-based method produced identical scores as did the CPU-based Tide and Tide-Hi.

**Conclusion:**

We propose the accelerating score function to search modifications using a single GPU. The software is available at https://github.com/Tide-for-PTM-search/Tide-for-PTM-search.

## Background

Peptide identification is one of the most important problems in proteomics. Tandem mass spectra (MS/MS) are generated by peptides cleaved from proteins and analyzed using database search methods to identify the peptides [1]. An XCorr score function is used by SEQUEST [2], which is the most popular software for peptide identification. First, SEQUEST generates theoretical spectra using database sequences, compares the theoretical spectra to an experimental spectrum (called the XCorr score function), and finds the sequence most similar to the experimental spectrum. Given that the XCorr score function is time-consuming, this score function was developed to improve performance capabilities. Most recently, the HiXCorr algorithm [3] was developed for high-resolution spectra and implemented in conjunction with Tide [4] and Comet [5], with these score function referred to as Tide-Hi and Comet-Hi, respectively.

However, database search tools using XCorr score functions are still slow because candidate peptides increase when PTMs are considered in the search. A multi-thread method exploiting CPU cores has been used to resolve this problem. Recently, studies of high-performance computing applications have used GPUs for parallelization. Using GPUs, Tempest [6] improved the classical SEQUEST XCorr score function and FastPaSS [7] accelerated the spectral library search method. CPUs and GPUs have different methods for data processing. The GPU is designed for the simultaneous execution of a single instruction on many threads. For this reason, it is a different problem to implement the XCorr score function for each tool using the GPU, though it is an efficient method as a single GPU generally has more cores than a single CPU. In this paper, we used the GPU to develop the score function derived by combining Tide’s XCorr score function and the HiXCorr algorithm.

## Implementation

Our method is implemented in C++ and NVIDIA’s CUDA (Compute Unified Device Architecture). It appropriately uses both the CPU and the GPU. The preprocessing step of the experimental spectra applies the HiXCorr algorithm using the CPU. Because the result using HiXCorr algorithm is a sparse vector that increases the time of the dot product step, this result is mapped to a full vector using the GPU (Mapping step). Each thread of the GPU processes a single bin of the full vector in the mapping step. After this step, using the CPU, our method extracts candidate peptide sequences (Extracting step); then, using the GPU, our method creates the theoretical spectra (Creating step), and takes the dot product between the experimental spectra and the theoretical spectra (Dot product step). In the creating step and dot product step, each block and each thread of the GPU processes a single candidate peptide and a single peak of the theoretical spectrum, respectively.

## Results

For high-resolution spectra analysis, MS data were generated by CPTAC (Clinical Proteomic Tumor Analysis Consortium). Peptide fragmentation was performed with the high-energy collision-induced dissociation (HCD) method. The data were acquired on a Thermo Q-Exactive instrument. The first fraction of the Com-pRef_Proteome_BI_2 was used; it consists of 33,223 MS/MS data. For low-resolution spectra analysis, HAP1 cell was used and peptide fragmentation was collision-induced dissociation (CID) [8]. Tandem mass spectra were acquired on a using a linear trap quadrupole (LTQ) Orbitrap Velos mass spectrometer (Thermo Fisher Scientific, Waltham, MA). The first fraction of first replicate (M411-A01-O156-HS-P4569–1 and M411-A01-O156-HS-P4569–2) was used; it consists of 25,528 MS/MS data (PreoteomeXchange identifier: PXD006614). The MS/MS data were searched against the SwissProt human-reference (released in July 2016) database. Our method is compared with Tide (Crux version 3.1) [9] and Tide-Hi on a machine with an Intel Core i7-7700 K CPU (4.20GHz), 32GB of RAM and an NVIDIA GeForce GTX 1080 8GB GPU under CentOS 7.

Tide is generally used with parameters for specific PTMs and, when many PTMs are used, the number of candidate peptides is increased. Table 1 shows that the number of candidate peptides is increased when CPTAC data are searched with various PTMs for maximum missed cleavages = 2, number of enzyme termini (NTT) = 2, and precursor tolerance = 0.1 Da (Dalton). Considering many PTMs, Tide is slow because of the increase in the number of candidate peptides. Recently, the Open Search method [10] using 500 Da for precursor tolerance has been proposed for blind search. If precursor tolerance = 500 Da, all PTMs for ±500 Da are considered for the database search. Actually, the precursor tolerance is the PTMs mass range. As such, we changed the precursor tolerance to increase the number of candidate peptides instead of considering PTMs. Table 2 shows that as the precursor tolerance increases, the number of candidate peptides increases for maximum missed cleavage = 2, NTT = 2.

**Table 1:** Average numbers of candidate peptides for various PTMs using CPTAC data

PTM	Average number of candidate peptides
Non-modified	1089.70
1 Oxidation (M)	1348.12
1 Oxidation (M) 1 Deamidation (NQ)	2769.45
2 Oxidations (M) 2 Deamidations (NQ)	3752.88
2 Oxidations (M) 2 Deamidations (NQ) 1 Phosphorylations (STY)	8616.11

**Table 2:** Average numbers of candidate peptides for various precursor tolerances using CPTAC data

Precursor tolerance	Average number of candidate peptides
0.1	1089.70
0.2	1537.49
0.5	1641.89
1	3275.70
2	6547.26
5	16,332.48
10	32,556.57
20	65,217.44
50	162,928.67

We compared our method with Tide and Tide-Hi. Fragment tolerance = 1 Da was used for low-resolution spectra (HAP1), fragment tolerance = 0.02 Da was used for high-resolution spectra (CPTAC), and the time of tide-search excluding the tide-index time was measured.

**Fig. 1:**
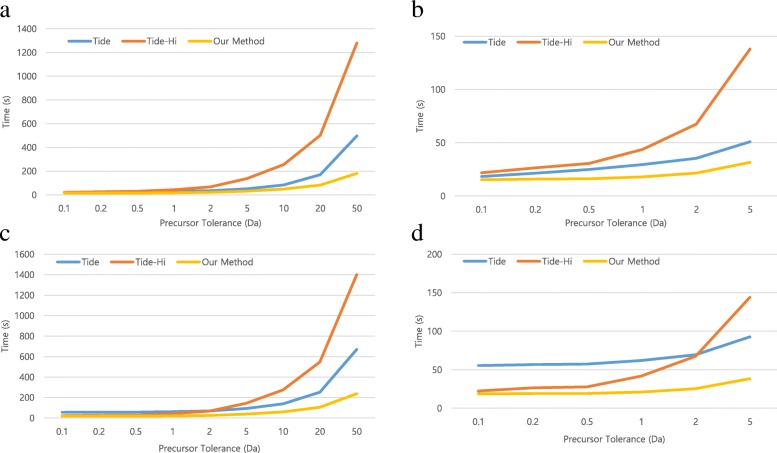
Comparison of total running time for Tide, Tide-Hi, and our method when 8-threads and various precursor tolerances were used. **a**, **b** Running time for low resolution spectra (fragment tolerance = 1 Da). **c**, **d** Running time for high-resolution spectra (fragment tolerance = 0.02 Da). **b** and **d** show enlarged results up to precursor tolerance = 5 Da in (**a**) and (**b**), respectively

When the number of candidate peptides is small, that is, when the precursor tolerance is narrow, Tide is faster than Tide-Hi for low-resolution spectra (Fig. 1 (a), (b)), but Tide-Hi is faster than Tide for high-resolution spectra (Fig. 1 (c), (d)), because Tide-Hi is implemented for high-resolution spectra. However, as the number of candidate peptides increases, Tide-Hi becomes slower than Tide. The time complexity of Tide is *O*(*n*) for preprocessing time and *O* (*mP*_*t*_) for calculated time of XCorr, where *n* is the size of the spectrum bin for the fragment tolerance, *m* is the number of candidate peptides, and *P*_*t*_ is the number of peaks in each theoretical spectrum. On the other hand, the time complexity of Tide-Hi is *O* (*P*_*e*_) for preprocessing time and *O*(*m* (*P*_*e*_ + *P*_*t*_)) for calculated time of XCorr, where *P*_*e*_ is the number of peaks in the experimental spectrum. If *m* (the number of candidate peptides) increases, *O* (*mP*_*e*_) becomes larger than *O*(*n*), so that Tide-Hi becomes slower than Tide. For this reason, Tide-Hi is slower than Tide as the number of candidate peptide increases.

**Fig. 2:**
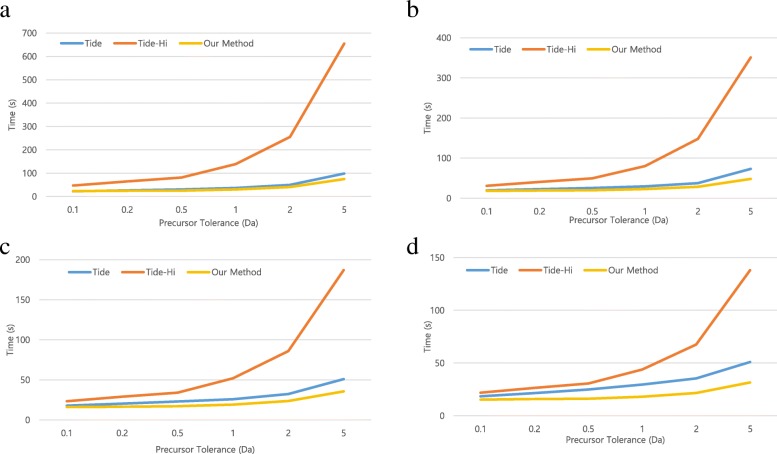
Comparison of total running time for Tide, Tide-Hi, and our method when various threads and low-resolution spectra (fragment tolerance = 1 Da) were used. **a** Single thread. **b** 2-threads. **c** 4-threads. **d** 8-threads

**Fig. 3:**
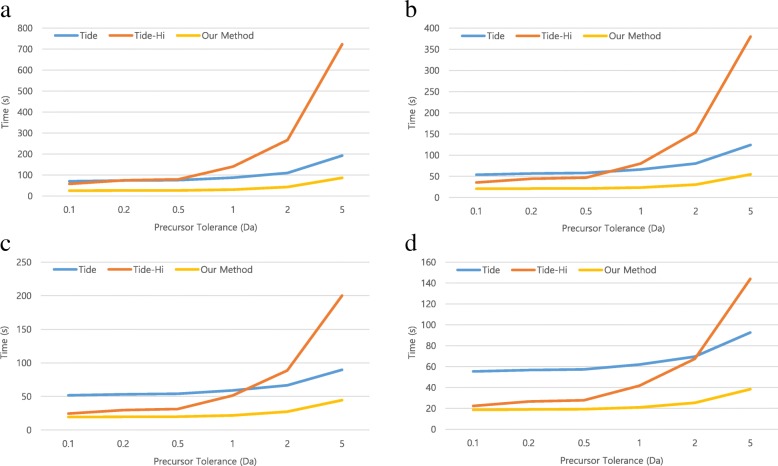
Comparison of total running time for Tide, Tide-Hi, and our method when various threads and high-resolution spectra (fragment tolerance = 0.02 Da) were used. **a** Single thread. **b** 2-threads. **c** 4-threads. **d** 8-threads

Our method, utilizing a single GPU, uses the HiXCorr algorithm to speed up the search for high-resolution spectra even as the number of candidate peptides increases. Figure 1 shows that the proposed method is faster than Tide-Hi and Tide even as the number of candidate peptides increases. Our method is faster than Tide and Tide-Hi regardless of the number of candidate peptides, or the resolution of the spectra. For low- and high-resolution spectra, our method is 2.7 and 5.8 times faster than Tide and Tide-Hi at a 50 Da precursor tolerance. Since Tide uses the multi-thread method, we measured the times by changing the number of threads. Figures 2 and 3 show that when using low- and high-resolution spectra, our method is faster than Tide and Tide-Hi, respectively, regardless of the number of threads. Our GPU-based method produced identical scores as did the CPU-based Tide and Tide-Hi.

## Conclusions

We propose an accelerating score function to search modifications using a single GPU. We used the GPU to develop the accelerating score function, which was derived by combining Tide’s XCorr score function and the HiXCorr algorithm. For low- and high-resolution spectra, our method is 2.7 and 5.8 times faster than the Tide and Tide-Hi for 50 Da precursor tolerance. The software is available at https://github.com/Tide-for-PTM-search/Tide-for-PTM-search.

## Availability and Requirements

**Project name:** Tide for PTM search.

**Project home page:** https://github.com/Tide-for-PTM-search/Tide-for-PTM-search

**Operating system(s):** CentOS 7.

**Programming language:** C++, CUDA.

**License:** Apache license.

**Any restrictions to use by non-academics:** none.

**Example data:** available at project homepage.

## Abbreviations

CPTAC: Clinical proteomic tumor analysis consortium
CUDA: Compute unified device architecture
Da: Dalton
GPU: Graphics processing unit
MS/MS: Tandem mass spectra
NTT: Number of enzyme termini
PTM: Post-translational modification
XCorr: Cross-correlation
